# Adenylate Kinase and Metabolic Signaling in Cancer Cells

**DOI:** 10.3389/fonc.2020.00660

**Published:** 2020-05-19

**Authors:** Aleksandr Klepinin, Song Zhang, Ljudmila Klepinina, Egle Rebane-Klemm, Andre Terzic, Tuuli Kaambre, Petras Dzeja

**Affiliations:** ^1^Laboratory of Chemical Biology, National Institute of Chemical Physics and Biophysics, Tallinn, Estonia; ^2^Department of Cardiovascular Medicine, Mayo Clinic, Rochester, MN, United States

**Keywords:** adenylate kinase, energy metabolism, phosphotransfer, mitochondria, cancer

## Abstract

A hallmark of cancer cells is the ability to rewire their bioenergetics and metabolic signaling circuits to fuel their uncontrolled proliferation and metastasis. Adenylate kinase (AK) is the critical enzyme in the metabolic monitoring of cellular adenine nucleotide homeostasis. It also directs AK→ AMP→ AMPK signaling controlling cell cycle and proliferation, and ATP energy transfer from mitochondria to distribute energy among cellular processes. The significance of AK isoform network in the regulation of a variety of cellular processes, which include cell differentiation and motility, is rapidly growing. Adenylate kinase 2 (AK2) isoform, localized in intermembrane and intra-cristae space, is vital for mitochondria nucleotide exchange and ATP export. AK2 deficiency disrupts cell energetics, causes severe human diseases, and is embryonically lethal in mice, signifying the importance of catalyzed phosphotransfer in cellular energetics. Suppression of AK phosphotransfer and AMP generation in cancer cells and consequently signaling through AMPK could be an important factor in the initiation of cancerous transformation, unleashing uncontrolled cell cycle and growth. Evidence also builds up that shift in AK isoforms is used later by cancer cells for rewiring energy metabolism to support their high proliferation activity and tumor progression. As cell motility is an energy-consuming process, positioning of AK isoforms to increased energy consumption sites could be an essential factor to incline cancer cells to metastases. In this review, we summarize recent advances in studies of the significance of AK isoforms involved in cancer cell metabolism, metabolic signaling, metastatic potential, and a therapeutic target.

## Introduction

**Figure 1:**
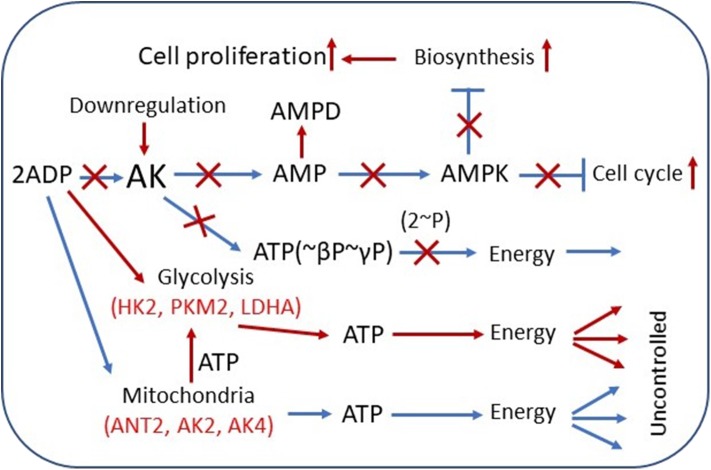
Overview of adenylate kinase (AK) isoform involvement in the rewiring of cancer cell metabolic signaling and energetic circuits. Increased competition for cytosolic ADP downregulates AK-mediated AMP signaling, reducing control over cell cycle and proliferation. AK expression is downregulated in several tumors. AMP can be consumed by AMPD and by 5′-NT, also overexpressed in some cancer cells. Augmented glycolytic metabolism, owing to higher affinity, scavenges cytosolic ADP, and uses mitochondrial ATP to drive glucose conversion to lactate. Overexpression of glycolytic HK2, PKM2, and LDHA and mitochondrial ANT2, AK2, AK4, and other genes in cancer cells promotes rewiring of energetic circuits resulting in unrestrained energy distribution. The result of these metabolic transformations is deficient AMP signaling and AMPK-mediated control of cellular katabolic and anabolic processes. Red color indicates the augmented pathways and gene expression in cancer cells. AMPD, AMP-deaminase; HK2, hexokinase 2; LDHA, lactate dehydrogenase A; ANT2, adenine nucleotide translocase 2; AMPK, AMP-activated protein kinase.

The significance of metabolism and metabolic signaling in human diseases is rapidly growing. New features and molecular players that are vital for cell homeostasis and function are being uncovered. Well-organized high-energy phosphoryl transfer systems are required to mediate intracellular communication between ATP-consuming and ATP-producing cellular compartments and thus to maintain normal growth and development of the cell (1–5). The main components of the cellular phosphotransfer system are AK, creatine kinase (CK), and glycolytic networks (1, 2). The significance of organized phosphotransfer was demonstrated by genetic manipulations in animal models, cellular systems, and alterations or mutations in separate phosphotransfer enzymes, which are associated with human diseases (6–16). Studies on *Drosophila* and mice model demonstrate that deletion of adenylate kinase 2 (AK2) is embryonically lethal, signifying the importance of AK phosphotransfer network in cell homeostasis (13, 17–19). In humans, mutations in the mitochondrial AK2 gene are associated with reticular dysgenesis characterized by immunodeficiency and sensorineural deafness, where processes of nucleotide signaling, cell differentiation, and motility are affected (15, 16, 20). So far, nine isoforms of adenylate kinase (AK1–AK9) and several subforms have been found and well characterized in mammalian cells (21). AK, which catalyzes reaction 2ADP↔AMP+ATP, is a recognized facilitator of AMP metabolic signaling, optimizing intracellular energetic communication, and local ATP supply (5, 22). Historically, the function of AK has been ascribed to *de novo* adenine nucleotide synthesis and cell energy economy through regulation of nucleotide ratios in different intracellular compartments and AMP-sensitive metabolic enzymes (14, 23, 24). The unique properties of AK lie on its ability to deliver γ- and β-phosphoryl groups of ATP, thereby doubling the ATP energetic potential. Moreover, the AK network provides an efficient mechanism for high-energy phosphoryl transport from mitochondria to ATP utilization sites (2). Evolutionary AK isoforms have been positioned to different subcellular compartments (21, 25). AK1, AK7, and AK8 are solely found in the cytosol; AK2, AK3, and AK4 are located in the mitochondria; and AK5 and AK9 can be found in either the cytosol or nucleus. Only AK1 and AK6 are known to be expressed in all tissues, whereas AK5 is expressed only in the brain (21). In the cytosol, the main isoform is AK1, which is predominantly expressed in high energy demand tissues such as the brain, heart, and skeletal muscles. AK2 is strategically located in the mitochondrial intermembrane and cristae space to facilitate high-energy phosphoryl exchange between mitochondria and cytosol (22). Two other AK isoforms, AK3 and AK4, are located in the mitochondrial matrix and are involved in the regulation of mitochondrial Krebs cycle and oxidative phosphorylation (OXPHOS), whereas AK5 and AK6 isoforms that are localized in the nucleus could serve to fulfill the energy needs of nuclear processes. In general, distinct intracellular localization and kinetic properties of AK isoforms favor energy support of specific cellular processes ranging from muscle contraction, electrical activity, cell motility, unfolded protein response, and mitochondrial/nuclear energetics (22). Importantly, reprogramming of energy metabolism has been proposed as one of the hallmarks of cancer (26), which is required to drive biosynthesis pathways necessary for rapid cell replication and proliferation. Cancer cells are believed to have a greater reliance on glycolytic phosphotransfer (27, 28). However, during the last decade, it was found that some tumors contain numerous mitochondria producing ATP predominantly *via* OXPHOS (29–31). The observed shift in hexokinase (HK) isoforms, upregulation of HK2 in cancer cells (32), indicates a closer integration of mitochondria with glycolytic phosphotransfer (see Figure 1). The association of HK2 with mitochondria and expression of pyruvate kinase PKM2 could promote effective yet uncontrolled energy distribution in cancer cells (27, 33, 34). Phosphotransfer enzymes such as CK and AK have been implicated in cancer cell proliferation (35, 36). However, it is not clear whether the redistribution of phosphotransfer enzymes, especially those which are localized in mitochondria, occurs during cancer formation. In this review, we focus on the significance of AK isoforms in the rewiring of cancer cell energy metabolism and AMP signaling. Specifically, we will overview how AK isoforms, localized in mitochondria (AK2 and AK4), and their main communication partners cytosolic AK (AK1 and AK6) are involved in cancer formation and metastasis.

## Adenylate Kinase 2 and Mitochondrial Creatine Kinase Interplay in Malignant Transformation

AK2 and mitochondrial CK (CKmit) are major phosphotransfer enzymes located in the intermembrane/cristae space in mitochondria (3, 14, 22). AK2 and CKmit provide nucleotide exchange and metabolic signaling capacity, allowing mitochondria to export ATP and reception of cytosolic feedback signals such as ADP, AMP, and creatine (22, 23, 37). Phosphotransfer enzymes CK and AK have been implicated in cancer cell proliferation (35, 36). In general, CK is involved in cancerous transformation, as CKB (brain-type CK) is upregulated in a variety of cancers to support growing energy needs (38). The elevation of creatine metabolites was noted in drug-resistant cancer cells (39). However, in other cancer types, the downregulation of CKB and rewiring of metabolism may play an important role in colon cancer progression (40). Moreover, several studies have demonstrated that in colorectal cancer (41), breast cancer (42), neuroblastoma (35), prostate cancer (43), and sarcoma (36, 44), the CKmit was downregulated. The reduction of CKmit in cancer cells was associated with the upregulation of adenylate kinase AK2 isoform in intermembrane space (36, 41, 42, 45, 46) (see Table 1). There is evidence that the expression of AK2 on the cell surface could facilitate nucleotide signaling and metastatic potential (60). It was found that Ak2 gene expression is upregulated in the metastatic pancreatic endocrine neoplasms (60), indicating the significance of nucleotide metabolic signaling in cancer invasion (61). Moreover, increased expression of the Ak2 on the surface of the metastatic F9DR murine terato-carcinoma cells compared with the nonmetastatic F9B9 cell line has been demonstrated (53). Furthermore, a recent study showed that AK2 has prognostic and therapeutic potential in lung adenocarcinoma (55). The knockdown of AK2 suppressed proliferation, migration, and invasion, as well as induced apoptosis and autophagy in human lung adenocarcinoma cells. In this regard, the AK2-FADD (Fas-associated protein with death domain) mediated apoptosis pathway was found to be defective in some tumor cells, which may contribute to tumor development by preventing apoptosis (62). A recent study indicates that AK2 and FADD are crucial for caspase-10 activation upon metabolic stress, and this activation is independent of death receptors and extrinsic pathway of apoptosis (63). Moreover, the deletion of the Ak2 gene or exit AK2 from mitochondria during apoptosis disrupts nucleotide exchange between mitochondria and cytosol, causing hyperpolarization of mitochondria and reactive oxygen species (ROS) production (20). It was found that the presence of AK2 in mitochondrial cristae nanochannels is critical for ATP export (2, 22). There are evidence that the AK2 upregulation could be used by cancer cells to support energy supply to biosynthetic processes and cellular growth (18, 64). These results, as well as studies on CK and AK knockout mice, demonstrate remarkable plasticity of cellular energetics and phosphotransfer systems, which could be used in cancer cells to promote uncontrolled cell growth (9, 17, 22, 65).

**Table 1:** Adenylate kinase isoforms in cancer.

**Enzyme**	**Type of cancer**	**Status in tumor**	**Localization**	**Function/therapeutic target**	**Experimental model**	**References**
AK	Lung cancer	↓	-	Negative regulator of cancer	Tissue samples	(47)
AK	Hepatomas	↓	-	Decreased during de-differentiation of cancer cells	Rat liver and hepatomas	(48)
AK	Colon cancer	↑	-	Metabolic regulator. Energy distribution shifts from CK toward AK	Tissue samples	(41, 49)
AK1	Transformed embryonic fibroblasts	↓	Cytosol	Negative regulator of tumor malignant	ras^V12^/E1A-transformed primary mouse embryonic fibroblasts	(50)
AK2	Breast cancer	↑	Mitochondria intermembrane space	Prognostic and therapeutic target	Estrogen receptor-negative breast cancer tissue samples	(51)
AK2	Breast cancer	↑	Mitochondria intermembrane space	Oncotarget of the breast CSC	Breast CSC	(52)
AK2	Breast cancer and neuroblastoma	↑	Mitochondria intermembrane space	Oncotarget of the poorly differentiated cancer cells	Tissue samples and cancer cell lines	(46)
AK2	Embryonic carcinoma	↑	Mitochondria intermembrane space	Metabolic regulator. Energy distribution shifts from CK toward AK	Cell lines	(36)
AK2	Teratocarcinoma	↑	Plasma membrane	Overexpressed on the plasma membrane in metastatic cells	Cell lines	(53)
AK2	Breast cancer	↓	Nuclear	Negative regulator of tumor cell growth via DUSP26/FADD signaling	Breast cancer cell lines and tissue samples	(54)
AK2	Lung cancer	↑	Mitochondria intermembrane space	Associated with poor survival of patients. Prognostic and therapeutic potential	Tissue samples	(55)
AK4	Lung cancer	↑	Mitochondrial matrix	Associated with poor survival of patients. Prognostic and therapeutic potential	Tissue samples and various cell lines	(56)
AK4	Glioma	↑	Mitochondrial matrix	A key regulator of intracellular ATP level. Prognostic and therapeutic potential	Tissue samples and cancer cell lines	(57)
AK6	Breast cancer Colon cancer	↑	Nuclear	Promote cancer cell growth. Prognostic and therapeutic potential	Colon adenocarcinoma and breast cancer tissues	(58)
AK6	Colon cancer	↑	Cytosol	Glycolysis regulator via phosphorylation LDHA. Modulator of CSC invasion and metastasis activity	CSC from tissues	(59)

## Adenylate Kinase Modulate Tumor Cell Response to Survive Under Oxidative Stress

The ability to conduct metabolic signaling and rewire metabolism is critical for cell survival. The AK4 isoform increased expression has been associated with a poor clinical outcome marker for lung cancer (56) as well as for glioma patients (57) (see Table 1). It was found that AK4 expression is under tight control of noncoding RNA. The AK4 is negatively regulated by micro-RNA miR-556-3p and positively by circular RNA of ATP-binding cassette (ABC) subfamily B member 10, circ-ABCB10 (66). In same study was demonstrated that downregulation of AK4 restrained lung cancer progression and sensitized lung cancer cells to cisplatin (66). Moreover, new data indicate that AK4 was shown to be involved in the radioresistance of esophageal cancer cells (67) and in chemoresistance of other cancers (68, 69). Previously, it was suggested that overexpression of AK4 could protect cells against oxidative stress (70). Other studies on HeLa (68) and HEK293 cells (71) demonstrated that tumor cells respond to a hypoxic condition by upregulating the AK4. However, in HepG2 cells (71), it was found that under oxidative stress, AK4 oppositely was downregulated. Although AK4 might be downregulated, it can still regulate OXPHOS because it retains the nucleotide-binding capability, and it can interact with the mitochondrial adenine nucleotide translocase (ANT) (70). It was found that knockout of AK4 increased cellular ATP through raised OXPHOS activity as well as mitochondrial number (68). Fujisawa and colleagues in 2016 have proposed that there are two mechanisms how AK4 regulates mitochondrial respiration in cancer cells (68). First, in cancer cells, AK4 interacts with ANT, which forms with voltage-dependent anion channel (VDAC) and HK transmembrane complex AK4-ANT-VADC-HK (see Figure 1). Under the hypoxic conditions, the complex supports the high glycolytic activity of cancer cells. It allows efficient ADP recycling between mitochondrial ATP synthesis and glucose phosphorylation by HK, which interacts with the mitochondrial outer membrane (MOM) (68, 72). In addition, in hepatoma cells (73), it was found that up to 50% of ATP is provided by intramembrane space located AK2 through VDAC binding to HK. Thus, AK2 may also be a member of the metabolic circuit channeling ADP–ATP in and out of mitochondria. The second mechanism is related to the fact that AK4 and AK3 have highly homologous sequences; therefore, they compete with each other for their substrates (68). According to this mechanism, AK4 interferes with AK3 action in supplying of the GDP required for the conversion of succinyl-CoA to succinate. That is why overexpression of AK4 in HeLa cells induces a decrease in Krebs cycle metabolites such as succinate, fumarate, and malate while glutamine and glutamate are increased. In several tumors, it was shown that the predominate substrate for mitochondria is glutamine (74). However, further studies are needed to confirm the role of AK4 in mitochondria and Krebs cycle substrate metabolism.

## Adenylate Kinase Network Role in Cancer Stem Cells

Traditional therapies against cancer, such as chemotherapy and radiotherapy, have many limitations. The limitation is due to systematic and local toxicity as well as drug resistance of small populations of tumor cells that have self-renewal properties. This small population of cells is called cancer stem cells (CSCs) (75). Previously, studies on CSC have shown that the cancer resistance for chemotherapy is related to increased OXPHOS in CSC. That is why a new generation of cancer chemotherapy could be targeted against pathways that interact with OXPHOS, such as the phosphotransfer system. Lamb and colleagues have shown on the breast cancer model that mitochondrial mass is a new biomarker of CSC, which have increased AK2 expression level (see Table 1) (52). In our previous study on neuroblastoma (NB) (46), which contains numerous CSC (76), and embryonal carcinoma cells (36), we also found that those cells have a high activity of AK2 (see Table 1). Moreover, another feature of CSC is that mitochondria are localized around the cell nucleus (77). There is evidence that AK2 can play an important role in communication between mitochondria and the nucleus (78). In another study using proteomic analysis of mouse teratocarcinoma cells (53), it was demonstrated that metastatic cancer cells have increased AK2 levels than have nonmetastatic cancer cells (see Table 1). As metastasis is related to cell motility, positioning of phosphotransfer enzymes to sites of increased energy consumption could be an important factor of tumor formation (59). The AK4 has been identified as a biomarker of metastasis in lung cancer (56, 79, 80). Overexpression of AK4 promoted lung cancer metastasis by enhancing hypoxia-inducible factor HIF-1 stability and epithelial-to-mesenchymal transition under hypoxia (79). Moreover, it was found that aferin-A could suppress AK4-HIF-1a signaling and may serve as a novel anti-metastatic agent in lung cancer (79). The AK4 was also implicated in breast and bladder cancers, where it promoted cell proliferation and invasion (81, 82). Furthermore, it was demonstrated that another AK isoform AK6 could affect colorectal cancer migration and invasion (59). Although significant progress has been made, at this time, the complete role of the AK system in cancer metastasis is still unclear. Moreover, the other reason why CSCs are drug resistant relates to the increased expression of ABC transporters in those cells (83, 84). The model for ABC transporters was proposed (85), which is based on ^31^P solid-state NMR spectroscopy, suggesting that intrinsic ATPase is coupled with AK activity where AK participates in ATP exchange. It is known that cytosolic and membrane-associated AK can regulate the activity of another ABC protein—K-ATP channel (86, 87). Nevertheless, the exact role of AK in supporting adenylate charge and function of ABC transporters in CSC remains unknown yet. In this respect, the ABC transporters are not unique proteins that possess both ATPase and AK activities; there are other proteins like AK6 (58), also known as transcription factor TAF9, human coilin interacting nuclear ATPase protein (hCINAP), and highly conserved DNA repair complex Rad50 (88).

## Paradoxes Regarding the Role of Adenylate Kinase in Tumor Formation

Cancer is a very complex and diverse phenomenon, including tissue specificity and different phases. Enzymatic changes can be different in the initial and advanced stages of tumor growth (89, 90). There are some contradictory studies where it was found that in lung cancer and hepatoma, AK was downregulated compared with that in normal tissue (47, 48) (see Table 1), whereas a recent study has shown that high expression of AK2 correlates with a worse prognosis for lung cancer patients (55) (see Table 1). In mouse embryonic fibroblasts, it was demonstrated that during their transformation into tumor cells, a significant reduction of AK1 expression occurs (50). More recently, the existence of AK1 additional gene product AK1β has been reported, and it is known that the AK1β expression level is regulated by p53 (91). In some cancers, p53 is mutated or suppressed. In this context, experiments on mouse embryonic fibroblast (50) have shown that during their transformation into tumor cells, augmentation of AK1 might be related to the downregulation of AK1β (see Table 1). Also, Kim et al. have postulated that AK2 is a negative regulator of tumor growth (54) (see Table 1). They demonstrated that in some cells, the AK2 localized not only in mitochondria but also in the nucleus, where it interacted with dual-specificity phosphatase 26 (DUSP26). This protein complex can dephosphorylate FADD leading to suppressed cell growth. They also suggested that AK2 downregulation was associated with breast cancer formation. In contrast, Speers and colleagues have found that AK2 is overexpressed in ER-negative breast cancer (51) (see Table 1). They proposed that AK2 should be a novel target for the treatment of ER-negative breast cancer. Indeed, a diterpene lactone neoandrographolide from extracts of the traditional medicinal herb *Andrographis paniculata* has been suggested to inhibit AK2 and have strong anticancer properties (92). Nevertheless, studies on human breast cancer and colorectal cancer demonstrated another AK isoform AK6 was overexpressed during cancer formation (58) (see Table 1). These data correlate with our previous studies on colorectal and breast cancers (41, 46) (see Table 1). It was also shown, that in both colon and breast tissues, AK6 is located not only in nuclear but also in the cytosol. However, only in cytosolic compartmentalized AK6 did expression level increase during tumorogenesis of breast and colorectal cancer cells (58) (see Table 1). They have found that AK6's main function is to regulate ribosome assembly and, consequently, protein expression and cancer cell growth. Recently, it was demonstrated that hCINAP or AK6 is a potent modulator of metabolic reprogramming by phosphorylating LDHA, a key player in cancer glycolysis (59) (see Table 1). Thus, AK isoform role can be different depending on cancer cell type and development stage.

## Adenylate Kinase-Mediated Amp Metabolic Signaling in Cancer Cells

In recent years, AK-mediated AMP signaling is emerging as one of the most versatile systems in the regulation of diverse cellular processes (5, 22, 93). Particularly, AMP signaling to AMP-activated protein kinase (AMPK) plays a critical role in adjusting ATP-producing and ATP-consuming processes (90, 94) (Figure 1). In several cancers, it has been demonstrated that AMPK, a master regulator of cellular energy homeostasis, possesses tumor suppressor function (95–97). In cells, AMPK activation/suppression is regulated via changes in cellular AMP levels. The principal activator of AMPK is the AK-catalyzed pathway, where it monitors cellular ATP–ADP balance and signals to AMPK by increased AMP cellular level. A recent study indicates that AK and AMPK cooperate to maintain cellular ATP levels (98). On the other end, AMP-deaminase (AMPD) and 5′-nucleotidase (5′-NT) suppress AMPK via decreasing AMP cellular levels (22, 99, 100). Moreover, the product of AMPD and 5'-NT reactions is adenosine, an immunosuppressive metabolite. At a high level in tumors, adenosine can promote cell growth, invasion, metastasis of cancer cells, and tumor immune evasion (101). Our previous work has demonstrated that in NB and heart adenocarcinoma cells HL-1, their mitochondrial permeability for AMP was increased than in healthy cells (46). It is known that AK2, which has unique localization in mitochondrial space, has a high affinity for AMP among AMP metabolizing enzymes. Therefore, it has been proposed that the AK2's primary function is to regulate intracellular AMP levels and to guard the cellular adenine nucleotide pool (22). Our study also suggested that cancer cells have a high level of AK2 (46) (Figure 1). Altogether, in cancer cells, most cellular AMP transport occurs via MOM where it is converted immediately to ADP and channeled into, maintaining a low cytosolic AMP concentration. Recent direct measurements of AK-mediated metabolic flux indicate that cancer cells have suppressed ATP β-phosphoryl energetics and AMP signaling, as indicated from ^18^O-labeling experiments demonstrating that highly aggressive breast cancer cells MDAMB231 have lower β-ATP[^18^O] turnover (AMP phosphorylation) than have the control MCF10A cells (Klepinin et al., in preparation). This could be due to the rewiring of energy metabolism and glycolytic takeover. Activated glycolysis usually suppresses AK metabolic flux apparently by scavenging ADP (102) (Figure 1). Suppression of AK phosphotransfer, AMP generation, and consequent signaling through AMPK could be the biggest culprit of a cancerous transformation of a cell (Figure 1). There is also evidence that other AMP removal pathway enzymes like AMPD2 as well as 5′-NT are upregulated in colorectal cancer (103, 104). In this regard, the 5′-NT expression in breast cancer depends on tumor estrogen receptor status, suggesting a coordinated network (105). Our previous work has shown that in several tumors, MOM permeability has also increased for ADP, which may be related with keeping an intracellular ADP level low (41, 42, 49, 106) (see Figure 1). It was found that not only AMP but also ADP can regulate the activity of AMPK (107). Further studies are needed to elucidate detailed mechanisms: (1) how increased MOM permeability for ADP and AMP and (2) raised expression of AMP metabolizing enzymes can regulate intracellular nucleotide levels and the activity of AMPK and (3) what the significance is of AMP metabolic signaling in cancer progression.

## Conclusions

The present review is a snapshot from recent AK studies that focused on the significance of AK network in energetics and metabolic signaling in cancer cells. Of the nine AK isoforms (AK1–AK9), four of them (AK1, AK2, AK4, and AK6) are involved in the progression of malignant transformation. Studies indicate that AK isoforms (AK1, AK2, AK4, and AK6) have an important role in the regulation of cancer cell metabolism, metabolic signaling, and cell migration and invasion. Moreover, at the initial stage, suppression of AK phosphotransfer and AMP generation and consequently signaling through AMPK by a variety of factors could be the biggest culprit of the cancerous transformation of a cell. Downregulation of AK→ AMP→ AMPK signaling can lead to the loss of control of cell cycle, growth, and proliferation. In the later stages, as emerging data suggest, cancer cells may use the shift in AK isoforms and other phosphotransfer enzymes to rewire their energy supply circuits to support proliferation and metastasis. Knockdown of overexpressed AK2 in human lung adenocarcinoma cells suppressed proliferation, migration, and invasion as well as induced apoptosis and autophagy. In this regard, a diterpene lactone neoandrographolide from extracts of the traditional medicinal herb *Andrographis paniculata* has been suggested to inhibit AK2 and has strong anticancer properties. Further studies that involve all AK isoforms have the potential to bring new understanding and novel therapeutic strategies targeting the AK isoform network to suppress growth and metastasis of cancer cells.

## Author Contributions

AK and SZ performed the study design, development of methodology, data analysis and interpretation, drafting of the manuscript, and critical revision. LK and ER-K analyzed and interpreted data and performed manuscript review. AT, TK, and PD performed the study conception, design, writing, and reviewing of the manuscript.

## Conflict of Interest

The authors declare that the research was conducted in the absence of any commercial or financial relationships that could be construed as a potential conflict of interest.

## Abbreviations

AK: adenylate kinase
CK: creatine kinase
CKmit: mitochondrial creatine kinase
CKB: brain-type creatine kinase
MOM: mitochondrial outer membrane
ANT: adenine nucleotide translocase
AMPK: AMP-activated protein kinase
CSCs: cancer stem cells
NB: neuroblastoma
VDAC: voltage-dependent anion channel
ABC: ATP-binding cassette
FADD: Fas-associated protein with death domain
HIF: hypoxia-inducible factor
hCINAP: human coilin-interacting nuclear ATPase protein
DUSP26: dual-specificity phosphatase 26
OXPHOS: oxidative phosphorylation
AMPD: AMP-deaminase
5′-NT: 5′-nucleotidase
LDHA: lactate dehydrogenase A
HK: hexokinase.
